# Missed Diagnosis in Alcohol‐Related Hepatocellular Carcinoma: A Call for Alcohol Consumption and Liver Disease Early Detection

**DOI:** 10.1111/liv.70923

**Published:** 2026-10-11

**Authors:** Victoria Mignot, Aurore Baron, Thomas Decaens, Charlotte Costentin

**Affiliations:** ^1^ Université Grenoble Alpes Grenoble France; ^2^ Institute for Advanced Biosciences (IAB), Université Grenoble Alpes, Inserm U1209, CNRS UMR5309 Grenoble France; ^3^ Service D'hépato‐Gastroentérologie et Oncologie Digestive, CHU Grenoble Alpes, Université Grenoble Alpes Grenoble France; ^4^ Centre Hepato Biliaire, Paul Brousse Hospital Inserm URM 1193, Paris Saclay University Villejuif France

**Keywords:** alcohol‐related liver disease, health inequalities, hepatocellular carcinoma, surveillance

## Abstract

Alcohol‐related hepatocellular carcinoma is an increasingly prevalent consequence of alcohol use and the often‐silent progression of alcohol‐related liver disease. A substantial proportion of patients with alcohol‐related hepatocellular carcinoma are diagnosed at an advanced stage, frequently because underlying liver disease has not been previously identified and surveillance has not been implemented. This review summarizes recent epidemiological trends and clinical features of alcohol‐related hepatocellular carcinoma, with a particular focus on the high proportion of undiagnosed cirrhosis and the challenges posed by alcohol use disorder, comorbidities and socioeconomic vulnerability. Strengthening collaboration between hepatology and addiction medicine, alongside population‐level strategies to reduce alcohol use, will be essential to enhance surveillance uptake and ultimately improve outcomes in alcohol‐related hepatocellular carcinoma.

## Key Points

- Alcohol‐related hepatocellular carcinoma is frequently diagnosed at an advanced stage.
- This is largely due to unrecognized cirrhosis and poor uptake of surveillance in patients with alcohol‐related liver disease.
- Earlier identification of alcohol consumption should prompt systematic assessment of liver disease.
- This approach could substantially improve hepatocellular carcinoma surveillance, access to curative treatments and patient outcomes.

**Abbreviations**
ALD: alcohol‐related liver disease
HCC: hepatocellular carcinoma
NITs: non‐invasive fibrosis tests

## 1 Introduction

Primary liver cancer is the third leading cause of cancer‐related mortality and the sixth most common cancer in terms of incidence worldwide [1]. In 2022, primary liver cancer was estimated to account for 865 269 new cases and 757 948 deaths. Hepatocellular carcinoma (HCC) accounts for nearly 90% of all cases of primary liver cancer, and its incidence has increased significantly in recent decades, notably due to the global rise in alcohol exposure and the metabolic risk factors for chronic liver diseases, such as diabetes and obesity [2, 3]. Alcohol‐related liver disease (ALD) accounts for about 30% of HCC cases globally [4].

The epidemiology of alcohol‐related HCC varies substantially across world regions shaped by differences in alcohol consumption patterns, viral hepatitis prevalence and the burden of metabolic risk factors [5, 6, 7]. In Europe, alcohol represents one of the leading etiological contributors to HCC, while in many Asian countries HBV remains the predominant driver, even as alcohol consumption and metabolic dysfunction account for an increasingly significant proportion of cases [4, 5, 8]. In North America, the rising prevalence of obesity, Type 2 diabetes and harmful alcohol use have fuelled a growing incidence of HCC associated with overlapping or mixed aetiologies [6]. Likewise, Latin America is experiencing an epidemiological transition, with alcohol‐related liver disease and MASLD emerging as major causes of HCC alongside persistent viral hepatitis in several countries [9, 10]. In contrast, in sub‐Saharan Africa and the Middle East/North Africa, chronic HBV and HCV remain the predominant aetiologies, although the contribution of alcohol‐related liver disease and metabolic dysfunction is increasing in several settings [11, 12]. Collectively, these regional disparities underscore the critical importance of developing surveillance and prevention strategies that are tailored to local epidemiological profiles and risk factor distributions.

In France, an estimated 11 658 new cases of primary liver cancer and 8905 deaths attributable to primary liver cancer were reported in 2023 [13]. Primary liver cancer ranks as the fifth leading cause of cancer‐related death and the twelfth most common malignancy by incidence [1, 14]. Despite a steady decline in per capita alcohol consumption since the 1950s, alcohol consumption remains the leading cause of HCC [4, 15, 16, 17]. A study conducted in 2017 using the French National Health Data System demonstrated that alcohol, either as a sole risk factor or in combination with at least one additional etiological risk factor, was implicated in more than 60% of cases [16].

It is increasingly recognized that, in contemporary clinical practice, a substantial proportion of patients with HCC present with overlapping liver disease aetiologies, most notably the co‐occurrence of alcohol use disorder and metabolic dysfunction. The recent evolution in steatotic liver disease nomenclature, including the introduction of MASLD and MetALD, further reflects the complexity of this etiological overlap and the frequent coexistence of metabolic and alcohol‐related liver injury [18]. For the purpose of this review, the term “alcohol‐related HCC” encompasses HCC arising in patients whether as the primary aetiology or in the context of concurrent liver disease risk factors.

Although several recent reviews have addressed the epidemiology and management of alcohol‐related HCC, the present review takes a distinct perspective by focusing on missed opportunities for early diagnosis along the continuum from alcohol use disorder to cirrhosis and HCC development. In particular, we highlight the high prevalence of unrecognized cirrhosis, barriers to surveillance implementation—spanning patient, provider and healthcare‐system dimensions—and the potential role of non‐invasive fibrosis assessment tools to enable earlier identification of at‐risk individuals. We further examine alcohol‐related HCC through the lens of health inequalities and multidisciplinary care, with a practical emphasis on improving real‐world detection and surveillance strategies.

## 2 Hidden Yet Major Role of Alcohol in the Epidemiology of Hepatocellular Carcinoma

Alcohol is an independent risk factor for the development of HCC [19, 20, 21, 22]. However, it is important to note that, unlike HBV infection or metabolic dysfunction—which are relatively easy to investigate and to attribute as risk factors for liver disease—alcohol consumption is more difficult to quantify. Assessing alcohol intake requires a dedicated clinical interview, the reliability of which depends on factors related to both the patient and the clinician [23]. Patient‐related barriers include underreporting driven by stigma or poor insight, while clinician‐related factors encompass the extent to which the encounter is conducted non‐judgmentally and the degree to which the clinician is trained and comfortable in addressing alcohol use disorders. Moreover, quantifying alcohol intake alone is insufficient; identifying alcohol use disorder is equally critical to ensure that patients receive appropriate and targeted support for the management of their condition. Validated screening tools have been developed to identify hazardous alcohol use and alcohol use disorder, including the Alcohol Use Disorders Identification Test (AUDIT) and its shorter version, the AUDIT‐C, both of which are widely recommended across international guidelines dedicated to alcohol‐related liver disease [23, 24]. Unfortunately, these tools remain largely underused among hepatologists and gastroenterologists [23] underscoring the urgent need for integrated addiction care within liver and gastrointestinal clinics—not only to provide patient‐centred, evidence‐based management of alcohol use disorders but also to foster a broader cultural shift towards more proactive and systematic alcohol risk assessment across hepatology and GI units. This underuse partly explains why the proportion of HCC cases attributable to alcohol has been consistently underestimated, in contrast with the objective alcohol consumption data reported by the World Health Organization (WHO). This underestimation was corrected in an epidemiological study published in 2018, showing that even in China the proportion of HCC cases attributable to alcohol reached 27.8%, compared to 53.8% for HBV [5].

The risk of HCC increases in a dose‐ and duration‐dependent manner with alcohol consumption; importantly, no level of consumption has been identified as entirely free of associated hepatic risk, and abstinence remains the only threshold at which this risk is absent [25]. Chronic alcohol consumption exceeding 80 g/day for more than 10 years increases the risk of HCC fivefold [26]. However, an increased risk of HCC has been reported for lower levels of consumption such as 25 g/day [27]. In a case–control study including 115 patients with HCC and 230 cancer‐free controls, alcohol consumption (≥ 80 g/day) was strongly and independently associated with HCC (aOR 4.5 [1.4–14.8]). Although the strongest associations were observed for chronic hepatitis C (aOR 15.3 [4.3–54.4]) and chronic hepatitis B (aOR 12.6 [2.5–63.1]), alcohol misuse ranked among the leading risk factors, alongside diabetes mellitus (aOR 4.3 [1.9–9.9]). Moreover, synergistic interactions were observed between alcohol consumption and chronic hepatitis virus infection (OR, 53.9 [7.0–415.7]) and diabetes mellitus (OR, 9.9 [2.5–39.3]) [28].

Chronic alcohol drives the development of ALD, a condition encompassing a histological continuum from reversible hepatic steatosis to fibrosis and cirrhosis. Cirrhosis, the most advanced stage, represents the primary risk substrate to alcohol‐related HCC. Notably, in patients who developed HCC in a non‐fibrotic liver, alcohol consumption was not independently associated with an increased risk of HCC after adjustment for smoking habits and metabolic comorbidities, suggesting that fibrosis stage is a key determinant of hepatocarcinogenic risk [29]. In patients with alcohol‐related cirrhosis, the annual incidence of HCC is approximately 3% [5, 30].

## 3 Social Inequalities and Alcohol‐Related HCC

It is important to highlight social disparities associated with alcohol‐related HCC. The contribution of alcohol to HCC aetiology varies depending on the region of the world, with notably higher prevalence in Eastern and Central Europe [5]. Marked geographical disparities may also exist within the same country. In France, a study leveraging hospital databases reported significant heterogeneity in geographical distribution of HCC, with incidence of alcohol‐related HCC in northern and western France being up to eightfold higher than HCV‐related HCC incidence [15]. Overall, HCC affects men more often than women, but the sex ratio varies depending on the prevalence of risk factors across world regions [6].

Accumulating evidence from European and North American studies suggests that alcohol‐related liver disease and HCC disproportionately affect socially disadvantaged populations [30, 31, 32, 33]. In several European studies, although alcohol consumption is generally more frequent and intense among populations with higher educational attainment, alcohol‐related mortality disproportionately affects those with lower education levels, precarious employment and economic insecurity [8]. In addition, European and North American studies have reported higher incidence and alcohol‐related HCC‐specific mortality rates among the most disadvantaged populations [8, 31, 32]. In Asia‐Pacific regions, alcohol consumption frequently coexists with viral hepatitis and metabolic risk factors, giving rise to complex, mixed etiological profiles. This etiological overlap not only complicates the attribution of HCC risk to alcohol specifically, but may also contribute to a gradual shift in the epidemiological profile of the disease across the region [34]. However, most available data regarding social inequalities in alcohol‐related HCC originate from European and North American cohorts, and caution is warranted when extrapolating these findings to other healthcare settings.

Unhealthy dietary patterns and lifestyle behaviours, exposure to environmental contaminants and inequitable access to preventive care, screening and specialized hepatology services may all contribute to the social disparities observed in the incidence and outcomes of alcohol‐related HCC [8].

Addressing these disparities requires that public health interventions be both universal in reach and proportionate in intensity—a principle sometimes referred to as *proportionate universalism*—whereby the scope and resources allocated to interventions are scaled according to the level of social disadvantage, in order to reduce health inequities without further stigmatizing vulnerable populations [35].

## 4 Detecting Alcohol Use Disorder and Reducing Harmful Consumption: Essential Preventive Interventions

The risk of developing cancer (HCC and other gastrointestinal cancers) decreases with abstinence in the general population [36]. It has been reported that more than 20 years of abstinence are required to achieve an incidence of HCC comparable to that observed in individuals without alcohol exposure [37]. In patients with cirrhosis, the impact of abstinence on HCC risk remains debated. In a cohort of 22 287 patients with alcohol‐related cirrhosis, with or without viral co‐infection, the risk of HCC was correlated with alcohol consumption and the duration of abstinence (< 5 years, 5–10 years, > 10 years). In patients with alcohol‐related cirrhosis alone (*n* = 1652), the risk of HCC decreased significantly after 5 years of abstinence (OR 0.37 [0.18–0.77] between 5 and 10 years; OR 0.27 [0.14–0.50] beyond 10 years). Similar results were observed in the groups with viral co‐infection [38]. On the other hand, a meta‐analysis including more than 15 000 patients with cirrhosis showed that abstinence significantly reduced the risk of liver decompensation (HR 0.61 [0.47–0.79], *p* = 0.002), but not HCC risk (HR 0.86 [0.62–1.20], *p* = 0.28) [39]. In another cohort of patients with alcohol‐related cirrhosis followed at a tertiary centre, persistent alcohol consumption was associated with HCC onset in univariate analysis (HR 1.98 [1.18–3.31]), but this association disappeared after adjustment (*p* = 0.36) [39]. Possible explanations for these discrepancies could be related to imbalance in population characteristics, particularly the severity of the underlying cirrhosis. A recent study involving 727 patients with alcohol‐related cirrhosis (including 480 decompensated) suggests that the impact of abstinence depends on the stage of the disease. Among abstinent patients (48.7%), incidence of HCC was 2.3 per 100 person‐years and abstinence was associated with a significant reduction in HCC risk (HR 0.35 [0.13–0.94]). However, this association was only observed in patients who had never experienced liver decompensation, after adjustment for competing risks of death [30]. Thus, lack of a protective effect observed in the most severe populations could be explained by the presence of competing mortality risks, masking the potential impact of abstinence on HCC occurrence. Importantly, even after alcohol consumption reduction or cessation in patients with advanced fibrosis, the risk of HCC may decrease but does not disappear, thereby requiring continued monitoring [30].

Alcohol cessation remains the major goal to preserve liver function, reduce the risk of HCC and to facilitate HCC management if HCC occurs. Screening and treatment of viral hepatitis, as well as the management of metabolic comorbidities, are also essential components of managing steatotic liver diseases and preventing the risk of HCC. Altogether, a substantial proportion of HCC cases could potentially be prevented in regions where alcohol is a major driver of liver disease, particularly through improved management of alcohol use disorder and promotion of abstinence [40].

## 5 Poor Prognosis Mainly Related to Late Diagnosis

Accumulating evidence supports that alcohol‐related HCC is most often diagnosed at an advanced stage, thereby limiting access to curative treatment and worsening prognosis. In a prospective study that included patients with newly diagnosed HCC between 2007 and 2009, alcohol‐related HCC was more likely to be detected at an advanced tumour stage in patients with impaired general condition and liver function compared to other aetiologies [41]. Median overall survival was significantly lower in patients with alcohol‐related HCC than in those with HCC of other aetiologies (5.7 vs. 9.7 months, respectively; *p* < 0.01). However, the prognostic impact of alcohol disappeared when survival was assessed separately according to tumour stage at diagnosis. Patients with HCC detected during a cirrhosis monitoring program had better survival compared to patients with incidentally diagnosed HCC (11.7 vs. 5.4 months; *p* < 0.001). In another study conducted in the French national hospital database system, patients with alcohol‐related HCC had a poorer prognosis compared to HCV‐related HCC. Alcohol‐related HCC was also less frequently detected through a cirrhosis monitoring program, confirming the critical role of cirrhosis follow‐up programs and early identification of underlying ALD [17]. Higher prevalence of extra‐hepatic comorbidities and more precarious social conditions of patients may be factors limiting access to HCC‐specific treatments [8], in particular to curative strategies.

Early detection of HCC in patients with cirrhosis is a cornerstone of national and international clinical guidelines, as timely diagnosis remains the most critical determinant of access to curative treatments and, ultimately, of overall prognosis [42, 43, 44]. Indeed, an early‐stage diagnosis is associated with eligibility to curative treatment strategies, which is linked to prolonged survival. The vast majority of alcohol‐related HCC cases develop on an established cirrhosis that precedes the onset of HCC by several years, providing the opportunity to implement a surveillance program [45]. A major challenge, however, lies in the fact that underlying cirrhosis frequently follows an asymptomatic course, and is often diagnosed only at the time of HCC detection—precluding any opportunity for surveillance‐based early diagnosis. In a study conducted in the United States using the Veterans Health Administration database, 25% of patients diagnosed with HCC had unrecognized cirrhosis before the cancer diagnosis. Patients with ALD were more likely to have a concomitant diagnosis of liver disease and cancer (OR 1.69, 95% CI 1.06–2.67) compared to patients with chronic HCV infection. Importantly, patients with unrecognized cirrhosis were 6.5 times more likely to have advanced‐stage HCC at the time of diagnosis, which is associated with poorer survival [46].

## 6 Promote HCC Surveillance to Improve Overall Prognosis of Alcohol‐Related HCC

International guidelines recommend semiannual HCC surveillance using liver ultrasound in patients with cirrhosis, a strategy associated with increased eligibility for curative‐intent therapies and improved survival [43]. An incidence threshold of 1.5% per year for HCC is considered necessary for the cost‐effectiveness of semiannual HCC surveillance [43]. Conflicting data on the incidence of HCC in patients with alcohol‐related cirrhosis have sparked controversy in the literature regarding the value of periodic HCC surveillance in these patients [21]. Nevertheless, prospective data from Western European cohorts indicate that 6‐monthly ultrasound surveillance in patients with compensated alcohol‐related cirrhosis is associated with an HCC incidence close to 3%, while enabling frequent detection of tumours at an early stage [30].

Regarding HCC surveillance, abdominal ultrasound carries well‐recognized limitations, including suboptimal sensitivity for early‐stage HCC detection, particularly in patients with poor acoustic windows such as those with obesity or advanced cirrhosis. Furthermore, the cost‐effectiveness of ultrasound‐based surveillance remains debated and is highly dependent on the underlying risk profile of the target population. Access to regular imaging surveillance is also unequal across healthcare settings, representing a significant barrier in resource‐limited environments. Finally, false‐positive findings are not uncommon and may trigger a cascade of unnecessary and costly diagnostic investigations, with potential psychological burden for patients. However, semi‐annual ultrasound remains the cornerstone of HCC surveillance, and every effort should be made to improve surveillance uptake. In Japan, the national surveillance program in at‐risk patients achieves a 70% curative treatment rate, which is a realistic goal given the tools exist [47].

Unfortunately, in clinical practice, surveillance uptake is poor [48, 49]. In France, it is reported that fewer than 30% of HCC cases are detected through regular follow‐up, resulting in a high rate (> 70%) of late‐stage diagnoses that are not eligible for curative treatment [41]. In a study conducted within the French National Health Data System, 38% of patients with newly diagnosed HCC received no cancer‐specific treatment after HCC diagnosis [50]. The reasons for low implementation of surveillance guidelines are multiple. According to a study conducted in the United States, responsibility for surveillance failure was evenly split between referring physician (lack of a prescription) and patients (failing to undergo the prescribed test) [51]. The prescription of surveillance ultrasound according to guidelines was more frequent if the patient's follow‐up was managed by a liver specialist, highlighting the importance of specialized monitoring for liver diseases eligible for HCC surveillance. Patients with ALD were more likely to experience a lack of prescription for the surveillance ultrasound. On the other hand, lower adherence to surveillance programs has been reported among patients with alcohol‐related cirrhosis, particularly in cases of ongoing alcohol consumption [51, 52, 53]. However, in a cohort of patients with cirrhosis prospectively followed within a strict surveillance program, prognosis was independent of cirrhosis aetiology and was mainly driven by liver function, tumour characteristics and treatment allocation (curative vs. non‐curative) [22]. These results suggest that ALD itself is not necessarily associated with poorer outcomes, but rather that failures in the diagnostic and surveillance pathway contribute substantially to the worse prognosis observed in alcohol‐related HCC, such as undiagnosed cirrhosis and inadequate surveillance prescription or adherence.

It is important to note that in this study, 85% of patients with alcohol‐related HCC were abstinent (68%) or had low residual consumption (< 10 g per day, 17%) at the start of the surveillance program, which may explain good adherence to surveillance recommendations compared to patients with active alcohol use [53]. Among the patient‐related reasons for not undergoing surveillance, patient awareness that cirrhosis is a risk factor for HCC has been reported to be associated with the completion of the prescribed surveillance examination [54]. In a study conducted on a cohort of 105 patients with cirrhosis, mostly related to alcohol (65%), the level of health literacy was low, highlighting the urgent need to provide interventions to improve patient information and promote their engagement in care, particularly in the HCC surveillance program [55].

Importantly, not all patients with alcohol‐related cirrhosis have the same risk of developing HCC, and the indication for surveillance could be refined in order to focus surveillance and monitoring efforts on the highest‐risk patients. It has been reported that annual HCC rates varied considerably according to patient characteristics, ranging from 0.3% in patients under 55 years old with a platelet count > 125 000/mm^3^ to 4.8% in patients over 55 years old [56]. Other variables such as male sex, obesity and levels of alpha‐fetoprotein or bilirubin were also associated with the risk of developing HCC in patients with alcohol‐related cirrhosis and could be useful to personalize HCC surveillance strategies [30]. Importantly, active alcohol consumption can modulate HCC risk over time in a dose‐dependent manner in individuals with advanced chronic liver disease. These data support the systematic and repeated assessment of alcohol use—not only at the time of ALD diagnosis, but on a regular basis throughout follow‐up—leveraging validated tools such as the AUDIT and AUDIT‐C as part of the semi‐annual disease check‐up. Furthermore, evolving patterns of alcohol consumption should be incorporated into risk stratification models aimed at tailoring HCC surveillance programs to individual risk profiles [57]. Although risk‐tailored surveillance intervals have not yet been incorporated into current clinical guidelines, several complementary approaches are currently under evaluation. Abbreviated MRI protocols have shown promising diagnostic performance while reducing examination time and costs, particularly in patients with suboptimal ultrasound examinations [58, 59]. Trials with abbreviated MRI focusing on high‐risk patients are ongoing (NCT05095714). In addition, validated blood‐based biomarker panels, such as the GALAD score, may complement imaging‐based surveillance in selected patients [60]. Together, these emerging approaches illustrate the ongoing transition towards more personalized HCC surveillance, although they are not yet recommended for routine clinical practice.

Although these emerging approaches hold promise for the future, the greatest opportunity to improve outcomes currently lies in increasing the uptake and quality of guideline‐recommended HCC surveillance. Several practical interventions may help improve the implementation of HCC surveillance in patients with alcohol‐related cirrhosis. At the healthcare‐system level, automatic reminders and electronic health record‐based prompts represent promising tools to facilitate both the prescription of surveillance and the tracking of follow‐up appointments. At the provider level, closer integration between hepatology, primary care and addiction medicine, through collaborative or co‐located care models, may enhance continuity of care and reduce missed opportunities for timely surveillance. Finally, at the patient level, structured educational interventions addressing cirrhosis and HCC risk, combined with nurse‐led outreach and navigation programs, may improve both surveillance adherence and broader health literacy. Although the evidence base supporting these interventions remains limited, their implementation may nonetheless help dismantle the multilevel barriers that continue to drive the underutilization of HCC surveillance in routine clinical practice [61].

## 7 Early Identification of Alcohol‐Related Liver Disease Is the Key

In patients with alcohol consumption, it is critical to assess liver health, particularly to identify patients with cirrhosis, who are eligible for semiannual HCC surveillance. Liver biopsy is the reference for evaluating the degree of liver fibrosis, but it is an imperfect test (sampling bias, inter‐pathologist reading variability) and not suitable for screening (invasive and costly technique). It is also important to emphasize that normal liver biochemical tests do not rule out cirrhosis [62]. Non‐invasive fibrosis tests (NITs) represent an opportunity to screen for liver fibrosis. First generation simple blood tests combine indirect biomarkers of fibrosis, whereas specialized tests integrate direct biomarkers of liver fibrosis. Simple tests (FIB‐4, NAFLD fibrosis score, eLIFT, Forns Index) have the advantage of being low‐cost and easy to use. Specialized blood tests (Fibrometer, Fibrotest, ELF) offer greater accuracy but at additional costs [63, 64, 65]. Diagnostic studies using liver biopsy as a reference have demonstrated good sensitivity and specificity of NITs for the diagnosis of advanced fibrosis [66]. However, it should be noted that data regarding the prognostic value of NITs in the specific context of ALD remain limited in the literature. A meta‐analysis published in 2021 highlighted the low number of studies specifically devoted to the evaluation of NITs in patients with ALD. However, the available data suggest that NITs show good prognostic performance in ALD [67].

EASL guidelines recommend assessing liver fibrosis in all patients with risk factors for steatotic liver disease, particularly alcohol consumption [68]. The sequential screening strategy is based on the FIB‐4 blood test, easily calculated online with age, ALT, AST and platelet counts. If FIB‐4 is ≥ 1.3, a second‐line test is recommended (FibroScan). If liver stiffness is ≥ 8 kPa, patients should be considered at risk of advanced liver fibrosis and referred to a liver specialist, who will assess whether semiannual HCC surveillance should be initiated. If FIB‐4 is below 1.3, or the FibroScan result is below 8 kPa, the risk of significant liver fibrosis is low, and specialized referral is not advised, but in the case of persistent risk factors (such as persisting alcohol abuse), it is nonetheless recommended to repeat FIB‐4 every 1 to 3 years, depending on the number of associated risk factors (the more risk factors are associated, the shorter the interval between assessments). Figure 1 illustrates a primary‐care sequential fibrosis assessment strategy applicable in primary care settings, not restricted to ALD patients, but extended to broader at‐risk populations to identify patients at risk of advanced fibrosis.

**FIGURE 1:**
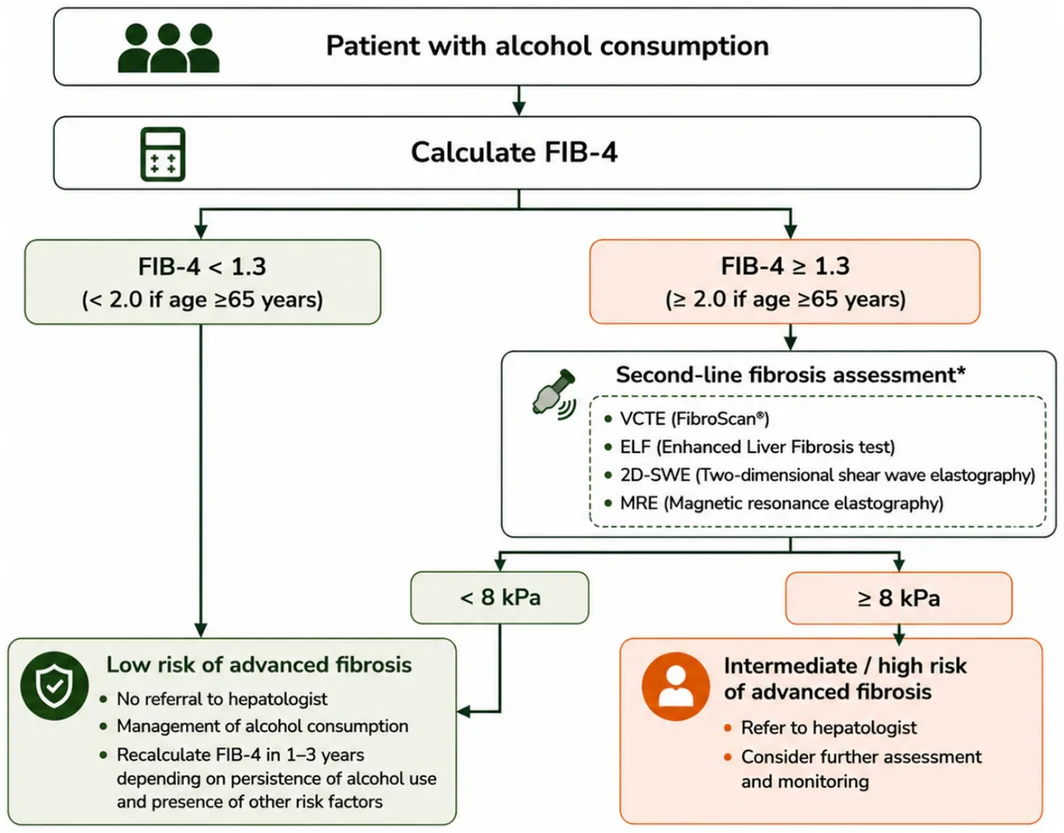
Proposed algorithm for sequential screening of liver fibrosis in primary care for patients with alcohol consumption. *VCTE (FibroScan) is the second‐line test recommended in the EASL sequential algorithm. Other validated second‐line fibrosis assessment techniques (ELF, 2D‐SWE and MRE) may be used according to local availability and expertise.

Although sequential FIB‐4 and transient elastography strategies are increasingly recommended for fibrosis risk stratification in routine clinical practice, several limitations should be acknowledged. First, both FIB‐4 and elastography‐based approaches are associated with intermediate or “grey‐zone” results that may complicate clinical decision‐making. In addition, their diagnostic performance may vary according to the underlying liver disease aetiology, including alcohol‐related liver disease, metabolic dysfunction‐associated steatotic liver disease and chronic viral hepatitis. Liver stiffness measurement is also operator‐dependent and may be influenced by obesity and technical factors. Specific caveats should also be considered in patients with harmful alcohol use. Active alcohol consumption and acute alcohol‐related hepatitis may increase AST levels and transiently elevate FIB‐4 values, while thrombocytopenia related to alcohol use may also influence test performance [68]. Similarly, liver stiffness values may be influenced by AST and bilirubin levels, active alcohol consumption and acute alcohol‐related hepatitis, potentially leading to overestimation of fibrosis stage [69]. Although current guidelines continue to endorse widely used non‐invasive fibrosis markers including FIB‐4 and liver stiffness thresholds, in patients with ALD, their interpretation warrants particular caution in the setting of active alcohol consumption or alcohol‐related hepatic inflammation, both of which may artifactually elevate fibrosis estimates. Non‐invasive fibrosis tests should therefore always be interpreted in light of the clinical context, and results obtained in patients with recent heavy alcohol consumption should be regarded as provisional, with reassessment recommended following an adequate period of alcohol withdrawal where clinically feasible.

## 8 Management Strategies for Alcohol‐Related HCC

### 8.1 Impact of Alcohol Consumption on HCC Treatments

Abstinence reduces the risk of hepatic decompensation in alcohol‐related cirrhosis and enhances eligibility for HCC‐specific treatments, which generally require preserved liver function (Child‐Pugh score A or B7) [43, 70]. In patients undergoing treatment for HCC, alcohol withdrawal has been associated with a significant reduction in overall mortality [71]. Furthermore, continued alcohol consumption has been linked to poorer oncological outcomes and reduced tolerance of both liver resection and transarterial chemoembolization [72, 73].

Data regarding the impact of ongoing alcohol consumption and abstinence on modern systemic therapies remain limited. Most available evidence is retrospective and based on subgroup analyses according to HCC aetiology. Some studies suggest that outcomes under immunotherapy‐based combinations such as atezolizumab‐bevacizumab may differ according to the underlying liver disease aetiology, although results remain inconsistent and data specifically evaluating active alcohol consumption during treatment are scarce [74, 75]. Experimental and translational studies have also suggested that alcohol exposure may promote resistance to sorafenib [76].

Finally, access to liver transplantation in the setting of HCC requires appropriate addiction management with a goal of abstinence. However, the duration of abstinence required for transplantation eligibility is not standardized. Overall, abstinence is therefore strongly recommended at the time of HCC diagnosis to improve HCC management and prognosis [77].

### 8.2 Aim for Curative Strategies

Curative treatment strategies for HCC are surgical resection, percutaneous ablation and liver transplantation and are associated with the best survival outcomes [43, 44]. Several studies have reported that alcohol‐related HCC was more likely to be detected at an advanced tumour stage in patients with impaired general condition and liver function resulting in reduced rates of curative treatment compared with other aetiologies [17, 41, 78, 79, 80].

However, prospective data from patients with compensated cirrhosis enrolled in a regular surveillance program demonstrated that HCC arising in the context of alcohol‐related cirrhosis can frequently be detected at an early stage, with access to curative treatments comparable to that observed in viral cirrhosis, despite a higher burden of comorbidities and more impaired liver function [22].

These findings suggest that reduced access to curative therapies in alcohol‐related HCC is primarily driven by delayed diagnosis and suboptimal surveillance implementation rather than by alcohol‐related aetiology per se.

### 8.3 Liver Transplantation for HCC: Specific Issues Related to the ‘Alcohol’ Context

Because liver transplantation represents a major curative option for early‐stage HCC, barriers to transplant access in patients with alcohol‐related liver disease may directly contribute to poorer oncological outcomes through delayed referral or tumour progression beyond established transplant criteria. However, most available data regarding transplantation outcomes and alcohol consumption relapse concern ALD overall rather than patients transplanted specifically for HCC arising in the setting of ALD.

Ethical and allocation concerns surrounding liver transplantation are particularly relevant as ALD has become the leading indication for liver transplantation in Europe, especially given the risk of post‐transplant alcohol relapse in the setting of organ shortage [81]. Negative perceptions regarding transplantation in ALD may also contribute to under‐referral of eligible patients to transplant centres [80]. Only 21% of patients with alcohol‐related cirrhosis who were eligible for transplantation were referred to a specialized transplant centre, highlighting persistent negative perceptions and barriers to referral in ALD [82, 83].

Although overall post‐transplant survival in ALD has historically been reported to be comparable to that observed in other aetiologies [84, 85, 86], sustained post‐transplant alcohol consumption is associated with poorer long‐term outcomes [87]. Importantly, occasional alcohol consumption appears to have a different prognostic significance than persistent or excessive drinking, which is more clearly associated with adverse outcomes [87].

Current practice increasingly relies on comprehensive psychosocial assessment aimed at identifying patients at higher risk of post‐transplant alcohol relapse rather than on a fixed duration of abstinence alone, as the predictive value of the historical 6‐month abstinence rule remains inconsistent across studies [88]. Factors associated with increased relapse risk include psychiatric comorbidities, limited social support, previous failed attempts at abstinence and family history of alcohol use disorder [88, 89, 90]. Accordingly, current EASL liver transplantation guidelines emphasize multidisciplinary evaluation and integration of addiction specialists within transplant teams [89, 91]. To achieve this goal, greater efforts should be made to improve the recognition of alcohol use disorders in post‐transplant care. Evidence suggests that addiction specialists outperform both the AUDIT‐C questionnaire and hepatologists in uncovering ongoing alcohol consumption, supporting the integration of structured pre‐ and post‐transplant addiction management as a means to reduce relapse risk and improve long‐term outcomes [91, 92]. Importantly, restoration of abstinence after post‐transplant alcohol use has been associated with post‐transplant survival comparable to that observed in patients without evidence of alcohol relapse, highlighting the importance of early relapse identification and structured addiction management before and after transplantation [93].

Liver transplantation practices in ALD are evolving, with increasing acceptance of early transplantation in selected patients with severe alcohol‐associated hepatitis and/or HCC without requiring prolonged pre‐transplant abstinence in all cases. While this approach may improve access to potentially life‐saving transplantation, it also raises concerns regarding the risk of post‐transplant alcohol relapse, further emphasizing the importance of careful psychosocial evaluation and multidisciplinary addiction management. However, the optimal strategies to balance access to transplantation with relapse risk assessment remain incompletely defined.

## 9 Closing the Diagnostic Gap in Alcohol‐Related HCC: Practical and Immediate Improvements to Routine Clinical Surveillance

Alcohol‐related HCC represents one of the most preventable forms of liver cancer. Given that its primary risk factor—alcohol consumption—is modifiable, primary prevention must remain the cornerstone of public health strategies targeting this malignancy. Effective alcohol control policies, early intervention for alcohol use disorder and population‐level health education are essential components of a comprehensive approach to reducing the future burden of alcohol‐related HCC. In routine clinical practice, alcohol intake should be systematically assessed using validated screening tools such as the Alcohol Use Disorders Identification Test (AUDIT) or its shorter version, AUDIT‐C [94, 95]. Although not specifically developed for MetALD, these tools are widely used in routine practice and may help identify patients with harmful alcohol use, including those within MetALD or ALD categories. A positive screen should prompt brief alcohol interventions and assessment for underlying liver disease, particularly through non‐invasive fibrosis evaluation when appropriate. Primary care providers are often the first point of contact for these patients and may therefore play a central role in prevention and early intervention. Dedicated awareness strategies, addressing alcohol‐related liver disease recognition, screening of at‐risk drinking behaviours and fibrosis risk stratification, represent a key lever to rationalize specialist referral and focus downstream evaluation on patients at significant risk of advanced fibrosis and HCC.

Because self‐reported alcohol intake frequently underestimates actual consumption, objective alcohol biomarkers may also help improve identification of patients with harmful alcohol use. Among these, phosphatidylethanol (PEth), a direct biomarker of alcohol consumption, has demonstrated good diagnostic performance for detecting recent and chronic alcohol intake and may help classify patients within the ALD or MetALD spectrum [96, 97, 98, 99]. This is even more interesting that emerging data also suggest that PEth may predict long‐term outcomes more accurately than self‐reported alcohol consumption in patients with ALD [100]. Although its integration into routine hepatology practice still requires further standardization, PEth may represent a promising complementary tool for risk stratification and longitudinal monitoring of alcohol use. Indeed, providing an objective, quantifiable measure of alcohol exposure that outperforms self‐report in predicting survival, PEth has the potential to refine HCC risk stratification, guide surveillance decisions and serve as a dynamic biomarker to inform clinical management across the full spectrum of alcohol‐related liver disease. Critically, the identification of alcohol use disorder should prompt referral to addiction specialists and the initiation of specialized care. Yet, in many healthcare settings, the resources necessary to support such pathways remain insufficient. Health authorities should therefore consider investing in specialized addiction care, in light of the considerable survival benefit associated with addiction management following severe complications of alcohol‐related liver disease [101].

Once harmful alcohol use is established, a major challenge remains the early identification of alcohol‐related liver disease prior to progression to cirrhosis and HCC. Failures at this stage operate at multiple levels, including limited awareness of liver disease risk among healthcare providers and insufficient implementation of fibrosis assessment strategies in routine care. In at‐risk populations, broader use of simple non‐invasive fibrosis tests such as FIB‐4 could facilitate the detection of previously unrecognized advanced fibrosis or cirrhosis. Automatic calculation and reporting of FIB‐4 scores by clinical laboratories have also been proposed as a potential strategy to facilitate implementation in routine practice, although its real‐world impact still requires further evaluation.

Even when cirrhosis is diagnosed, substantial gaps persist in the real‐world implementation of HCC surveillance programs. Failures occur at multiple levels: at the patient level, through poor adherence and limited health literacy; at the provider level, through under‐prescription of surveillance; and at healthcare‐system level, through fragmented care pathways and inadequate coordination. A considerable proportion of individuals with alcohol‐related liver disease remain undiagnosed until advanced stages, further delaying surveillance initiation and contributing to late‐stage HCC presentation. Furthermore, patients with alcoholic hepatitis often fall outside structured follow‐up pathways, reflecting the need for improved linkage between acute care episodes and long‐term surveillance. Importantly, surveillance should not be limited to the detection of HCC alone. Systematic screening for fatty liver disease and alcohol consumption throughout the patient's disease trajectory offers a critical opportunity to identify high‐risk individuals before malignant transformation occurs, thereby shifting the clinical paradigm from reactive diagnosis towards proactive, prevention‐oriented care.

Optimal management of alcohol‐related HCC requires a multidisciplinary approach, integrating hepatology, oncology, addiction medicine, nutrition and psychosocial support (Figure 2). This holistic model ensures that both tumour control and the underlying alcohol use disorder are addressed concurrently, improving survival and quality of life. Finally, addressing alcohol‐related HCC from the perspective of proportional universalism—that is, providing preventive and diagnostic interventions to all, but with intensity proportionate to need—may help bridge the current inequalities in care. Populations with higher alcohol‐related disease burden, social deprivation, or limited healthcare access should receive targeted reinforcement of prevention, early detection and comprehensive management strategies.

**FIGURE 2:**
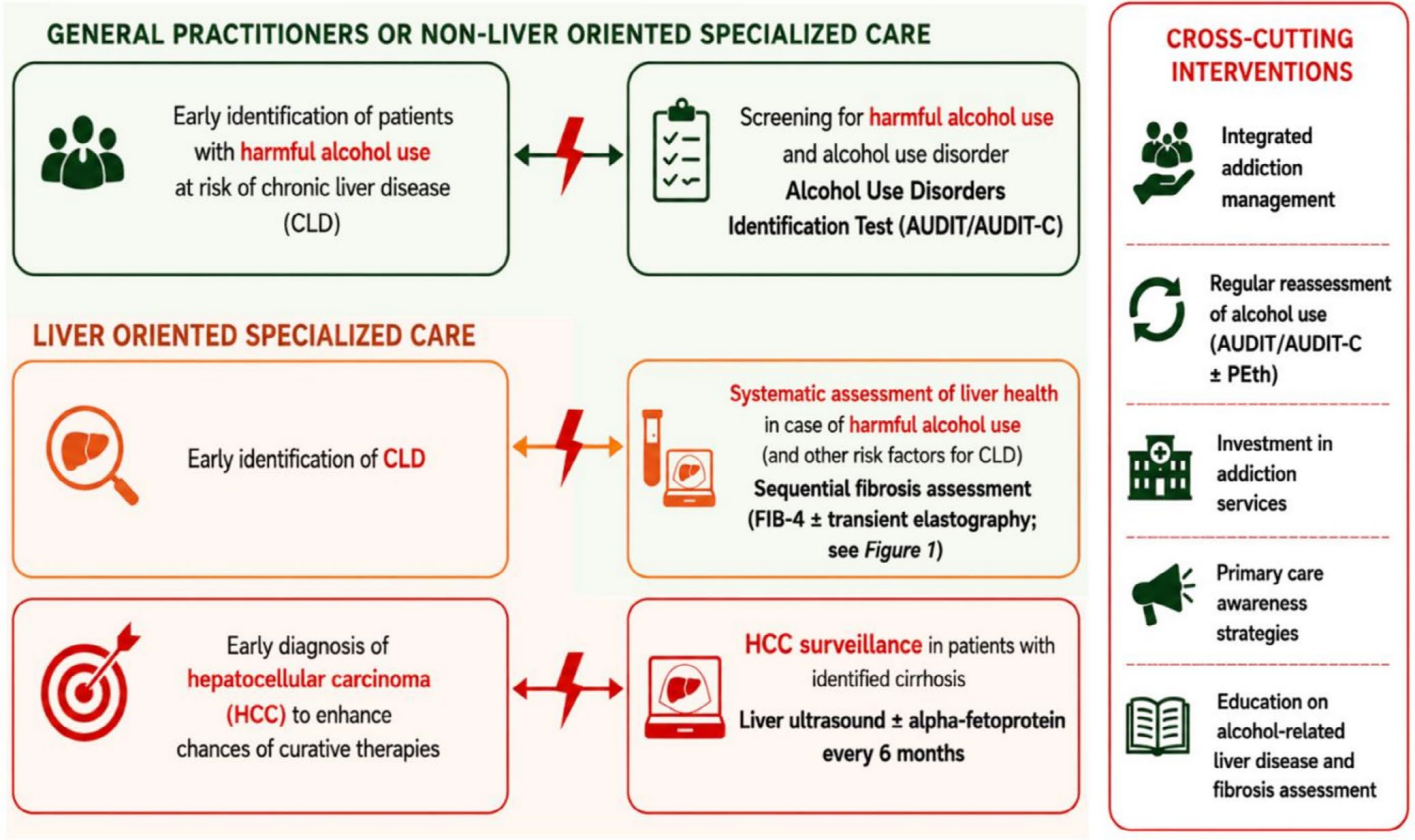
Gaps in the screening process for alcohol‐related liver disease and proposed strategies for improvement.

## 10 Conclusion

In conclusion, reducing the incidence and impact of alcohol‐related HCC demands a dual focus on prevention and early detection, supported by equitable, multidisciplinary care. By combining population‐level alcohol control with proportionate, patient‐centred strategies, the trajectory of this preventable cancer can be meaningfully altered. The key barriers and practical solutions discussed throughout this review are summarized in Table 1, providing a practical framework to support earlier diagnosis and more effective HCC surveillance in routine clinical practice.

**TABLE 1:** Multilevel barriers to the early diagnosis and surveillance of alcohol‐related hepatocellular carcinoma: towards actionable strategies.

Level	Barriers	Actions to improve early diagnosis and surveillance
Patient‐related	Low awareness of cirrhosis and HCC risk Low health literacy	Patient education
	Under‐reporting of alcohol consumption	Routine alcohol screening (AUDIT/AUDIT‐C) Integration of addiction care
	Poor adherence to surveillance	Reminder and navigation programs
Physician‐related	Failure to identify advanced fibrosis/cirrhosis	Sequential fibrosis assessment (FIB‐4 followed by second‐line tests)
	Under‐prescription of HCC surveillance	Clinical reminder systems
	Limited knowledge of guidelines	Guideline dissemination and education
	Delayed referral to hepatologist	Multidisciplinary care pathways
Healthcare system‐related	Limited access to hepatologist or imaging	Structured surveillance programs at the national level Promote liver health as a public health priority
	Fragmented care pathways	Integrated care pathways
	Absence of recall systems	Electronic reminder systems
	Socioeconomic disparities	Public health policies reducing inequalities
Future perspectives	Heterogeneous HCC risk among patients with alcohol‐related cirrhosis	Development of risk‐stratified surveillance, abbreviated MRI and blood‐based biomarkers to complement ultrasound in selected patients (not yet recommended for routine practice).

## Author Contributions

**Victoria Mignot:** investigation, writing – original draft, writing – review and editing. **Aurore Baron:** investigation, writing – original draft, writing – review and editing. **Thomas Decaens:** investigation, writing – review and editing. **Charlotte Costentin:** conceptualization, investigation, supervision, writing – original draft, writing – review and editing. All authors critically reviewed and approved the final version of the manuscript.

## Funding

The authors have nothing to report.

## Conflicts of Interest

The authors declare no conflicts of interest.

## Data Availability

Data sharing not applicable to this article as no datasets were generated or analysed during the current study.
